# Rehab-DRLX: explainable neurorehabilitation prognosis using deep reinforcement learning and transformer-based models

**DOI:** 10.3389/fncom.2026.1808274

**Published:** 2026-05-18

**Authors:** Hadeel Alsolai, Shakir Khan, Rakesh Kumar Mahendran, Arvind Panwar, Bayan Ibrahimm Alabduallah, Fatimah Alhayan

**Affiliations:** 1 Department of Information Systems, College of Computer and Information Sciences, Princess Nourah Bint Abdulrahman University, Riyadh, Saudi Arabia; 2 Information Technology Department, College of Computer and Information Sciences, Imam Mohammad Ibn Saud Islamic University (IMSIU), Riyadh, Saudi Arabia; 3 Department of Computer Science and Engineering, Saveetha Engineering College, Chennai, India; 4 School of Computer Science and Engineering, Galgotias University, Greater Noida, UP, India

**Keywords:** Explainable artificial intelligence (XAI), brain computer interface (BCI), deep reinforcement learning (DRL), neurorehabilitation, transformer

## Abstract

Neurorehabilitation poses a crucial problem in clinical recovery tasks, particularly for individuals with poor motor functions and neurological impairments, and problems in activities of daily living (ADL). To resolve this, we design a novel model, Rehab-DRLX, with a hybrid deep learning (HDL) framework that combines deep reinforcement learning (DRL) with an explainable transformer model to provide interpretable, accurate prognostic results. The propounded model is designed to effectively process the multimodal data inputs, which include clinical records, sensor-entrenched motion data, and neuroimaging, along with time-dependent recovery patterns from its reinforced representation learning (RRL) module. The RRL module employs a convolutional neural network (CNN) within the DRL agent, which performs spatiotemporal feature encoding and dynamically recovers a policy from its reward-guided learning method. To ensure interpretability, the explainable prognosis transformer (XPT) is utilized, which contains clinical contextual positional encoding and a hierarchical attention mechanism to enable transparent and reliable decision-making. This duality in the Rehab-DRLX architecture enables effective forecasting of the recovery outcomes, including functional independence probability, with both interpretability and accuracy, addressing the drawbacks of conventional black box prognosis tools. The experimental results of Rehab-DRLX show the noteworthy improvements in metrics such as accuracy (94.6%), F1-score (0.93), root mean square (RMSE) (0.082), and mean absolute error (MAE) (0.061) compared to existing studies. The ablation studies reveal the significant contribution of every architectural component and its overall performance. The results show the practical viability of Rehab-DRLX, which not only improves decision-making but also builds clinical trust through explainable insights.

## 1 Introduction

The neurorehabilitation field has shown noteworthy advancements over the past decades due to the convergence of deep learning algorithms and sensor-entrenched motion tracking (Alt Murphy et al., 2024). Those developments demonstrate its capability in intelligent healthcare systems, which not only effectively examine and monitor the movements but also forecast the trajectory recovery with higher precision (Tosto-Mancuso et al., 2022). During the physiotherapy session, with the help of wearable sensors and depth cameras, the patients' movements are captured at higher resolution, producing spatiotemporal data (Bonanno et al., 2023; Ferri et al., 2024; Ezra Tsur and Elkana, 2024). The adopted data are composed of multiple modalities, such as color imagery, depth mapping, joint orientation, and three-dimensional (3D) skeletal motion, providing an effective digital representation of patient rehabilitation activity (Sîrbu et al., 2022). That information paved the way for a new, personalized rehabilitation approach in which machine learning (ML) models can effectively identify recovery patterns and support adaptive treatment planning (Nguyen et al., 2024). However, the change from raw data sensor to actionable prediction poses a significant challenge in terms of variability, complexity, and uncertainty (Tsiamalou et al., 2022).

The major challenge in neurorehabilitation is its linear and adaptive nature. Every patient's recovery is effectively shaped by a combination of behavioral, neurological, and biological factors that interact unpredictably (Calderone et al., 2024). Some of the variables include psychological factors, rehabilitation exercise consistency and intensity, neuroplasticity, age of patients, and brain injury severity (Gentile et al., 2024). Conventional methods include statistical models and therapist examinations; therapists faced challenges in capturing recovery curves and fluidity. The adoption of machine learning enhances prediction outcomes but relies on shallow learning and hand-crafted features that do not capture temporal dependencies (Maurer-Karattup et al., 2022; Bartolo et al., 2023; Hoh et al., 2024). The use of deep learning is far superior to machine learning models, but it has a “black box” nature that affects the transparency and interpretability, thereby limiting the reliability and accountability of clinical decision-making (Jin et al., 2024; Gupta, 2024).

To resolve the gap between clinical applicability and technicality, the next-generation prognosis system has been developed in various critical ways. Initially, the next-generation system must learn from multimodal and high-dimensional data, including biometric signals, motion vectors, and joint trajectories without the need for manual features (Elashmawi et al., 2024). Second, the model must be adaptable to the patient's rehabilitation status. Third, the next-generation prognosis system must have the capability to effectively operate in a real-time environment, offering timely support during therapeutic sessions (Tripathi et al., 2025; Hemanth, 2023). After all those requirements, the main thing is that they must provide interpretable and explainable decisions to clinicians, which helps ensure shared decision-making and clinical validation. Although there have been many advancements in deep learning with attention mechanisms, some state-of-the-art approaches combine them with transparency and adaptability (Choo and Chang, 2022). On the whole, it was noted that there is a crucial need for a neurorehabilitation framework that not only utilizes deep learning but is also designed with ethical transparency, usability, and clinical interpretability (Sengupta et al., 2024).

By addressing existing issues through dynamic intelligence, interpretable reasoning, and adaptive learning, the proposed model, named Rehab-DRLX, combines deep reinforcement learning with Explainable artificial intelligence via a novel architecture. The Rehab-DRLX is designed to process forecast recovery outcomes and multimodal sensor data, thereby firmly aligning the therapeutic goals and clinical decision-making. It also utilized convolutional neural networks (CNN), which combined with the deep reinforcement learning (DRL) for capturing the kinematic and spatial features by also employing patient trajectory features over time. Those reinforced representations are provided to the transformer-enabled attention encoders to effectively highlight the prognosis trends, features, and key dependencies. More importantly, the Rehab-DRLX also combines a dual supervision strategy that effectively identifies a trade-off between the attention-entrenched explainability and the prediction accuracy, guaranteeing that the outcomes not only provide precision but also provide transparent clinical logic. With the hybrid approach, the proposed Rehab-DRLX aims to design a novel and noteworthy neurorehabilitation prognosis that offers a powerful, trustworthy tool that can learn, adapt, and explain patient conditions and recovery planning. The major contributions of this study are listed as follows:

- RRL for dynamic prognosis modeling: the proposed Rehab-DRLX introduces a noteworthy combination of CNN with DRL that ensures adaptive spatiotemporal learning from multimodal neurorehabilitation inputs. The reinforced representation learning (RRL) model enables firm recovery predictions entrenched in the longitudinal therapy and environment-based agent feedback.
- Trusted clinical prediction from XPT: the proposed framework also includes a transformer-enabled, explainable module, which is armed with contextual positional encoders and hierarchical attention gates for ensuring granular and interpretable prognosis decision-making that enables clinicians to effectively trace the therapy stages, sensor metrics, and specific neural events.
- Dual-supervision-enabled joint learning: with the dual loss function, the Rehab-DRLX is trained, which firmly optimizes the attention-based interpretability and recovery score prediction. With this bidirectional training objective, clinical decision-making becomes trustworthy and transparent.

## 2 Literature survey

(Bijalwan et al. 2023) propose a post-stroke rehabilitation (PSR) framework using a hybrid deep learning (HDL) model that combines a convolutional neural network and a bidirectional long short-term memory (LSTM). Using multi-layer representation learning, this study extracts temporal dependencies in lower limb exercises. However, this study hinders the adoption of clinical interpretability (i.e., not incorporating explainability methods). (Shao et al. 2023) design a prognosis prediction framework for brain–computer interface (BCI) from deep neural networks (DNNs) and rehabilitation signals. This study employs a domain-specific mechanism by focusing only on cortical patterns. However, its applicability is severely limited, which hinders adaptability across rehabilitation timelines. (Bunterngchit et al. 2025) design a motor imagery classification framework using a hybrid deep learning mechanism comprising a convolutional neural network and a gated recurrent network for stroke rehabilitation from electroencephalogram (EEG) signals. This study employs attention and inter-subject generalization techniques to amplify classification accuracy. However, the absence of a clear, well-defined prognosis severely limits the model's reliability when focused solely on classification tasks. (Zendehbad et al. 2025) present a hybrid transformer deep learning framework for providing assistive rehabilitation from EMG signals. With the help of deep sequence learning, this study effectively captures the temporal muscle movements which amplify assistive device control. However, due to its single-modality approach, this study lacks adaptability. (Zhao et al. 2025) design a motor imagery decoding framework by combining a transformer and a convolutional neural network. Temporal dependencies and multi-resolution spatial features are extracted using transformer and convolutional neural networks. Whereas, it hindered its performance in real-time applicability and longitudinal recovery relative to forecasting.

(Lin et al. 2022) designed a stroke recovery prognosis model using transfer-learning-enabled deep learning across varied rehabilitation modalities. This model combines personalized training data and domain adaptation to amplify generalizability across the patient population. It hinders dynamic recovery modeling due to poor feedback mechanisms and interpretability. (Miao et al. 2021) developed a machine learning model with a multimodal-sensor-enabled rehabilitation framework for stroke survivors in the upper limb. More specifically, their study integrates grip force sensors, surface EMG, and inertial sensors to achieve real-time feedback and categorization. However, it did not demonstrate long-term prognostic capability due to its shallow learning models. (Sohn et al. 2021) developed a brainstem auditory mechanism for stroke recovery BCI using machine learning models trained on electrophysiological signals. Although this study employs machine learning for intelligence, it struggles to capture the complex brain signal patterns, resulting in greater complexity. (Li et al. 2021) effectively examine the brain MRI features for the neurological rehabilitation process to predict the prognosis of cerebrovascular disease patients. They utilized structural imaging biomarkers for treatment planning and disease recovery. However, this system lacks behavioral and kinematic data. (Gao and Mao 2021) design an EEG-signal-enabled active rehabilitation model to assist stroke patients in cognitive recovery. This model effectively intervenes in dynamic EEG signals for cognitive assistance, whereas it lacks the ability to combine multiple sensors.

(Islam et al. 2022) utilize conventional machine learning algorithms and commodity sensors to examine neurorehabilitation exercises. At that point in therapy, the system employs inertial and kinetic sensors to assess motion accuracy. At the same time, it hinders the adoption of deep learning algorithms and does not capture complex spatial and temporal patterns. (Sandhu et al. 2024) developed machine-learning-based cognitive treatment planning for patients suffering from neurological disorders. This study effectively mapped cognitive metrics to rehabilitation trajectories. The sensor-based real-time approach was not included, resulting in poor feature learning. (Pirasteh et al. 2024) designed a rehabilitation control mechanism for amyotrophic lateral sclerosis (ALS) patients using EEG and neural signals. With the help of signal processing and categorization techniques, the assistive commands were provided. However, it did not apply to broader neurological applications with poor interpretability. (Lin et al. 2024) design an explainable deep learning model for forecasting motor recovery by employing BCI data and multi-state recovery fusion methods. More specifically, this study integrates attention-based convolution layers for motor-state prediction but lacks a long-term optimization. (Mukherjee and Roy 2024) developed a sensor-based finger rehabilitation technique for stroke patients from EEG sensors. During motor exercises, the brain signals are interpreted, providing precise, real-time assistance.

(Aslan and Yilmaz 2024) designed an EEG-signal-based dysphagia rehabilitation system by combining both deep learning and machine learning techniques. The neural signals can be effectively discriminated, thereby enabling the use of tongue-based features for rehabilitation. (Sayilgan 2025) employed spinal cord-injured patients in a BCI-adopted manifold learning framework. With the help of a non-linear dimensionality technique, the feature extraction process is achieved. However, it lacks real-time responsiveness. (Zhu et al. 2023) effectively designed a rehabilitation glove for BCI using deep learning for stroke patients. The gloves effectively interpret EEG signals and sync with user intention, providing immediate feedback, but lack long-term therapy planning. (Mishra et al. 2021) enhanced rehabilitation outcomes using machine learning by combining hand-grip actions with object recognition. Due to a lack of temporal modeling and continuous feedback, this study falls short. (Zhan et al. 2022) developed a rehabilitation training framework for chronic stroke patients for BCI. With the help of functional connectivity indices, the neurological effectiveness is measured, but it lacks in combining predictive deep learning for better forecasting.

## 3 Rehab-DRLX framework

### 3.1 Data acquisition system

**Figure 1:**
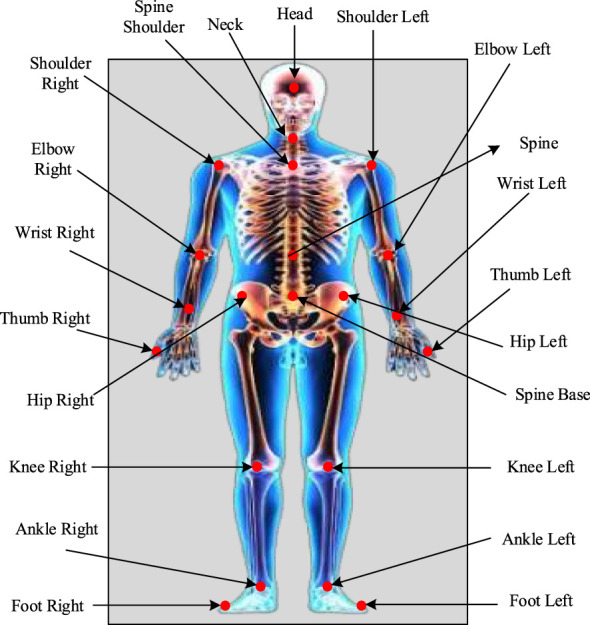
Illustration of skeletal joints by the Kinect v2 sensor.

**Figure 2:**
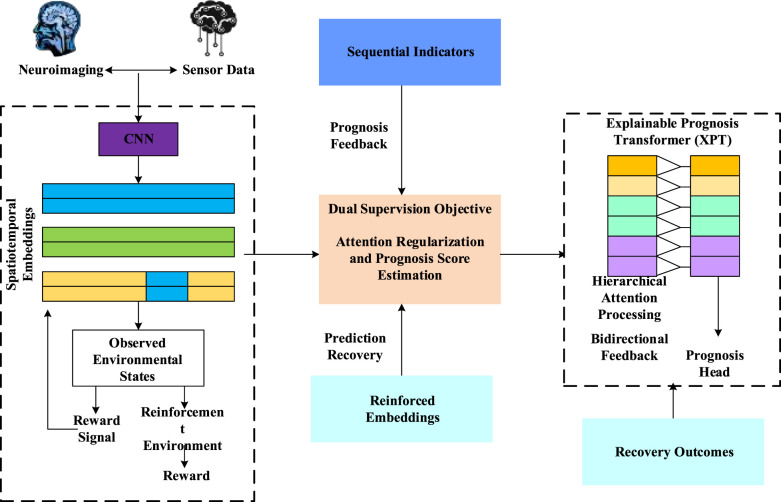
Overall architecture of the proposed Rehab-DRLX model.

To facilitate better neurorehabilitation, this study employs depth-sensing image technologies, namely, the Intel RealSense Depth camera D435 and Microsoft Kinect v2, which deliver markerless, non-invasive motion extraction capabilities. To validate and develop the proposed Rehab-DRLX framework, the Kinect v2 was employed, combining a red, blue, and green with depth channel D (RGB-D) camera and an RGB camera that concurrently extracts depth and color information. The Microsoft Kinect v2 sensor was used to extract complete skeletal data from patients' frontal views during physiotherapy sessions. To be clearer, we track 25 different human skeletal joints in real time. The patients are informed to be perform rehabilitation exercise Infront of Kinect sensor in which every joint was recorded along with modalities includes two-dimensional (2D) color image mapping (x,y), quaternions based joint orientations (x,y,z,w), 2D planar depth data (x,y), and RGB camera adopted 3D spatial coordinates (x,y,z). Figure 1 shows the illustration of skeletal joints tracking by the Kinect v2 sensor, whereas Figure 2 shows the overall architectural pipeline of the proposed Rehab-DRLX model.

### 3.2 Data acquisition method

In the laboratory setting of the Microsoft Kinect v2 sensor, the dataset for this study was collected under proper and consistent environmental conditions. With proper therapeutic relevance and advanced guidance, the rehabilitation exercises, particularly targeting lower limbs, were recorded effectively. More clearly, the dataset is composed of 25 voluntary participants with 10 normalized physiotherapy exercises, which consists of 10 females and 15 males. The age range for the participants spans from 20 to 60 years to guarantee demographic variability. For each time stamp, the Kinect v2 records four types of skeletal joints, as provided in the above subsection. The sensor operated at about 100 Hz, which provided higher temporal resolution for extracting fine-grained motion patterns. On the whole, the multimodal dataset provides a solid foundation for training a deep reinforcement-learning-enabled prognostic system with an explainable transformer.

The ground-truth prognosis labels were derived from therapist-guided rehabilitation performance scores and clinically relevant recovery indicators that reflect the quality and progression of therapeutic movements. These annotations served as the reference outcomes for training and evaluating the prognosis model. To ensure reliable model evaluation and to avoid potential data leakage across temporal sequences, a subject-wise cross-validation strategy was adopted during experimentation. Specifically, a five-fold cross-validation protocol was implemented, with participants divided into five mutually exclusive groups. In each fold, data from four groups were used for model training, while the remaining group was reserved exclusively for testing. This subject-wise partitioning ensures that motion sequences from the same participant do not appear simultaneously in both training and testing datasets. In addition, each rehabilitation exercise sequence was treated as a complete temporal trajectory, and temporal ordering was strictly preserved during preprocessing. By maintaining participant-level separation and temporal integrity of motion sequences, the proposed framework effectively prevents data leakage across time steps. It ensures a fair evaluation of the Rehab-DRLX model.

To define the prediction objective of the proposed framework, this study models rehabilitation outcome as a functional recovery score, reflecting the probability of achieving functional independence during therapeutic movement execution. The ground-truth labels were derived from clinician-guided rehabilitation assessments conducted during the experimental sessions. Specifically, physiotherapy experts evaluated the quality of each rehabilitation exercise based on factors such as joint coordination, smoothness of motion, balance stability, and completion accuracy. Each rehabilitation trial was assigned a recovery score within a normalized range of 0–1, where higher values indicate better functional movement quality and closer alignment with clinically expected rehabilitation performance. The annotation process was performed by multiple physiotherapy experts who independently reviewed the motion sequences and skeletal trajectories recorded by the Kinect v2 sensor. To ensure annotation reliability, the final ground-truth score used for model training and evaluation was determined by consensus among the clinical evaluators. This scoring protocol provides a clinically meaningful representation of rehabilitation progress and serves as the target variable for the prognosis prediction model. The predicted output of the Rehab-DRLX framework, therefore, represents the estimated functional recovery probability derived from multimodal motion and clinical features.

Within each fold's training set, 10% of the subjects were further allocated to a validation set to tune model parameters and prevent overfitting. In addition, the rehabilitation recordings were treated as complete temporal sequences, and no subsequences from the same recording were split across different dataset partitions. This approach preserves the temporal structure of rehabilitation trajectories and prevents temporal leakage between the training, validation, and testing datasets.

### 3.3 Reinforced representation learning module

The reinforced representation learning (RRL) module is responsible for spatiotemporal embedding optimization based on multimodal neurorehabilitation data. The RRL incorporates a convolution neural network (CNN) with deep reinforcement learning (DRL) in which the rehabilitation trajectory of every patient was modeled as a Markov decision process (MDP). Let the multimodal input data be denoted as


$$\begin{array}{l} {\text{ψ}}_{\text{t}}=\left\{{\text{y}}_{\text{t}}^{\text{img}},{\text{y}}_{\text{t}}^{\text{sen}},{\text{y}}_{\text{t}}^{\text{clin}}\;\right\},\text{ t}=1,2,\ldots ,\text{T} \end{array}$$ (1)

From Equation 1, the neuroimaging data at time t is denoted by ${\text{y}}_{\text{t}}^{\text{img}}\in {\text{ℝ}}^{\text{ℍ}\times \text{W}\times \text{ℂ}}$, the sensor-entrenched motion data extracted at the t-th time is denoted by ${\text{y}}_{\text{t}}^{\text{sen}}\in {\mathbb{R}}^{\text{d}}$, and the indicators of clinical trajectory are denoted by ${\text{y}}_{\text{t}}^{\text{clin}}\in {\text{ℝ}}^{ℸ}$. Those inputs are provided to the CNN for spatial feature extraction as


$$\begin{array}{l} {\text{z}}_{\text{t}}={\text{f}}_{\text{CNN}}\left({\text{ψ}}_{\text{t}}\right)\in {\text{ℝ}}^{⋗} \end{array}$$ (2)

From Equation 2, the *f*_*CNN*_(.) denotes the convolution encoder, and the t-th time encoder spatial feature vector is denoted by *z*_t_.

The rehabilitation timeline is modeled as a fine-horizon MDP, which is defined by a tuple in Equation 3,


$$\begin{array}{l} \text{Λ}=\left(\text{S},\text{A},\text{P},\text{R},\text{ζ}\right) \end{array}$$ (3)

where the state space is denoted by *S* that encoded embeddings as *s*_*t*_ = *z*_t_, the action space is denoted by A that provide recovery decision and treatment recommendations, the probability of transition is denoted by *P*(*s*_*t*+1_|*s*_*t*_, *a*_*t*_) that shows the temporal dependency in the progression of rehabilitation, the reward function is denoted by *R*(*s*_*t*_, *a*_*t*_) that computes the predicted recovery enhancement, and the discount factor is denoted by ζ∈[0, 1] that balances the short-term and long-term rewards. In the proposed reinforcement learning formulation, the action space does not directly correspond to explicit clinical treatment recommendations. Instead, the action represents the latent policy update performed by the reinforcement learning agent to refine the learned representation of rehabilitation trajectories. The DRL agent employs a neural policy network π_θ_(*a*_*t*_, *s*_*t*_) that is parameterized by θ as defined in Equation 4 for maximizing the expected cumulative rewards as


$$\begin{array}{l} \text{J}\left(\text{θ}\right)={\text{𝔼}}_{{\text{π}}_{\text{θ}}}\left[\sum_{\text{t}=1}^{\text{T}}{\text{ζ}}^{\text{t}}{\text{r}}_{\text{t}}\right] \end{array}$$ (4)

The reward *r*_t_ is calculated entrenched on two terms as


$$\begin{array}{l} {\text{r}}_{\text{t}}=\text{α}\cdot \text{Acc}\left(\text{t}\right)+\text{β}\cdot \text{Align}\left(\text{t}\right) \end{array}$$ (5)

From Equation 5, the prognosis prediction accuracy at the t-th time step is denoted by *Acc*(*t*), the clinical recovery scales for similarity validation are denoted by *Align*(*t*), and the balanced weights are denoted by α and β. The weighting coefficients α and β control the contribution of prognosis prediction accuracy and clinical recovery scale similarity in the reward formulation. In this study, these parameters were determined through empirical validation experiments rather than predefined clinical priors. Several candidate weight combinations were evaluated during model training to identify a balanced trade-off between prediction performance and alignment with clinical recovery indicators. The weights were constrained so that α+β = 1 to maintain normalized reward contributions. Based on validation results, the optimal configuration was α = 0.6 and β = 0.4, which enabled stable reinforcement learning convergence and improved prognosis consistency with clinical recovery assessments. The policy gradients are computed by employing actor–critic algorithms as


$$\begin{array}{l} \nabla_{\text{θ}}\text{J}\left(\text{θ}\right)={\text{E}}_{{\text{π}}_{\text{θ}}}[\nabla_{\text{θ}}\log{\text{π}}_{\text{θ}}\left({\text{a}}_{\text{t}}|{\text{s}}_{\text{t}}\right).{\hat{\text{A}}}_{\text{t}}] \end{array}$$ (6)

From Equation 6, the advantage function is denoted by ${\hat{\text{A}}}_{\text{t}}$ that computes how the action took place. Equation 7 formulates the final output of the RRL module is to update the embedding sequence as


$$\begin{array}{l} {\text{Z}}^{\text{RRL}}=\left\{{\text{z}}_{1}^{*},{\text{z}}_{2}^{*},\ldots ,{\text{z}}_{\text{T}}^{*}\right\} \end{array}$$ (7)

where the latent state, which DRL refines, is denoted by ${\text{z}}_{\text{t}}^{*}$ for adaptively optimizing to maximize the performance of the prognosis. Those embeddings are provided to the explainable prognosis transformer (XPT) for interpretable decision making.

### 3.4 Explainable prognosis transformer module

The XPT module serves as an interpretable decision layer in the Rehab-DRLX, accepting reinforced embeddings and clinical sequences. It effectively outputs prognostic predictions and provides transparent attribution of outcomes via dual supervision and a hybrid attention mechanism. There are two key inputs received by the XPT module, which are reinforced spatiotemporal embeddings from the RRL module and raw sequential clinical indicators as


$$\begin{array}{l} {\text{Z}}^{\text{RRL}}=\left\{{\text{z}}_{1}^{*},{\text{z}}_{2}^{*},\ldots ,{\text{z}}_{\text{T}}^{*}\right\},\;{\text{z}}_{\text{t}}^{*}\in {\text{ℝ}}^{\text{m}} \end{array}$$ (8)

$$\begin{array}{l} \text{C}=\left\{{\text{c}}_{1},{\text{c}}_{2},\ldots ,{\text{c}}_{\text{T}}\right\},\;{\text{c}}_{\text{t}}\in {\text{ℝ}}^{\text{k}} \end{array}$$ (9)

The two inputs expressed in Equations 8, 9 are combined and projected linearly to a unified model space as


$${\text{h}}_{\text{t}}{\text{=W}}_{\text{z}}{\text{z}}_{\text{t}}^{*}{\text{+W}}_{\text{c}}{\text{c}}_{\text{t}}\text{+b},\;{\text{h}}_{\text{t}}\in {\mathbb{R}}^{\text{d}}\;$$ (10)

From Equation 10, ${\text{W}}_{\text{z}},{\text{W}}_{\text{c}}\in {\text{ℝ}}^{\text{d}\times \text{m}},$ and ℝ^d × k^ are learnable projection matrices and *b*∈ℝ denotes the bias. The hierarchical attention encoding is denoted by $\text{H}=\left\{{\text{h}}_{1},{\text{h}}_{2},\ldots ,{\text{h}}_{\text{T}}\right\}\in {\text{ℝ}}^{\text{T}\times \text{d}}$. Those sequences are effectively processed from a standardized transformer encoder (Equation 11),


$$\begin{array}{l} \text{Att}\left(\text{Q},\text{K},\text{V}\right)=\text{sof}{\text{t}}_{\max}\left(\frac{\text{Q}{\text{K}}^{\text{T}}}{\sqrt{{\text{d}}_{\text{k}}}}\right)\text{V} \end{array}$$ (11)

For every head, the *Q* = *HW*_*Q*_, *K* = *HW*_*K*_, and *V* = *HW*_*V*_ with learnable matrices denoted by ${\text{W}}_{\text{Q}},\;{\text{W}}_{\text{K}},\text{ and }{\text{W}}_{\text{V}}\in {\text{ℝ}}^{\times_{ℸ}}$. The attention head outputs are combined and fed to the feed-forward block. To preserve clinical and temporal context, the position encoding matrix is defined as *P*∈ℝ^T × d^ and the clinical prior vector *e*_*ctx*_. Equation 12 outlines the output for the transformer is denoted by


$$\begin{array}{l} \tilde{\text{H}}=\text{H}+\text{P}+{\text{e}}_{\text{ctx}} \end{array}$$ (12)

More clearly, the transformer layer output is denoted in Equation 13,


$$\begin{array}{l} {\text{H}}^{\text{out}}=\text{TransEncoder}\left(\tilde{\text{H}}\right)\in {\text{ℝ}}^{\text{T}\times } \end{array}$$ (13)

This research also applies temporal attention as defined in Equation 14 for enabling the importance of every timestep by providing weight as,


$$\begin{array}{l} {\text{α}}_{\text{t}}=\frac{\text{exp}\left({\text{u}}^{⊺}\text{tanh}\left({\text{W}}_{\text{a}}{\text{h}}_{\text{t}}^{\text{out}}\right)\right)}{\sum_{\text{t}=1}^{\text{T}}\text{exp}\left({\text{u}}^{⊺}\tanh\left({\text{W}}_{\text{a}}{\text{h}}_{\text{t}}^{\text{out}}\right)\right)},\;\sum_{\text{t}}{\text{α}}_{\text{t}}=1 \end{array}$$ (14)

The context vector is computed by (Equation 15),


$$\begin{array}{l} \text{v}=\sum_{\text{t}=1}^{\text{T}}{\text{α}}_{\text{t}}\cdot {\text{h}}_{\text{t}}^{\text{out}} \end{array}$$ (15)

For prognosis prediction, the context vector is utilized for generation as


$$\begin{array}{l} \hat{\text{y}}=\text{σ}\left({\text{W}}_{\text{o}}\text{v}+{\text{b}}_{\text{o}}\right) \end{array}$$ (16)

From Equation 16, the recovery outcome, which is predicted, is denoted by $\hat{\text{y}}\in \text{ℝ}$, the sigmoid function is denoted by σ, and output weights are denoted by ${\text{W}}_{\text{o}}\in {\mathbb{R}}^{\text{1}\times \text{d}},\;{\text{b}}_{\text{o}}\in \;\mathbb{R}$.

### 3.5 Dual supervision objective

The XPT is trained using a dual loss function, the prognosis-and-explainability-aware loss function. In terms of the prognosis loss function, the ground-truth label *y* is denoted in Equations 17 and 18,


$${\text{L}}_{\text{pred}}\text{=}\frac{\text{1}}{\text{N}}{\left(\hat{{\text{y}}_{\text{j}}}-{\text{y}}_{\text{j}}\right)}^{\text{2}}$$ (17)

$$\begin{array}{l} {\text{L}}_{\text{pred}}=-\frac{1}{\text{N}}\sum_{\text{j}=1}^{\text{N}}{\text{y}}_{\text{j}}\log\left(\hat{{\text{y}}_{\text{j}}}\right)+\left(1-{\text{y}}_{\text{j}}\right)\log\left(1-\hat{{\text{y}}_{\text{j}}}\right) \end{array}$$ (18)

In terms of explainability-aware attention loss, the interpretability and sparsity in attention weights are encouraged as (Equation 19),


$$\begin{array}{l} {\text{L}}_{\exp}=\forall .\sum_{\text{t}=1}^{\text{T}}{\text{α}}_{\text{t}}\text{log}\left({\text{α}}_{\text{t}}\right) \end{array}$$ (19)

The entropy-entrenched regularizer penalizes uniformly distributed attention for key-moment focusing. The overall loss function is denoted in Equation 20,


$$\begin{array}{l} {\text{L}}_{\text{total}}={\text{L}}_{\text{pred}}+{\text{L}}_{\exp} \end{array}$$ (20)

where the regularization weight is denoted by ∀.

### 3.6 Ethical considerations

The dataset used in this study involves motion capture recordings from human participants performing rehabilitation exercises under controlled laboratory conditions. Prior to participation, all individuals were informed about the purpose of the study, the data collection procedures, and the intended research usage of the recorded motion data. Written informed consent was obtained from all participants before the experimental sessions. The data collected from the Microsoft Kinect v2 sensor consists solely of skeletal joint coordinates and motion trajectories, which do not contain any personally identifiable information. To protect participant privacy, all datasets were anonymized prior to processing and analysis. The study procedures followed standard research ethics and data governance guidelines for human participant research, and all collected data were used solely for academic research on neurorehabilitation analysis. These measures ensure that the study adheres to ethical principles concerning participant consent, privacy protection, and responsible data handling.

## 4 Experimental results

This section examines the performance of the Rehab-DRLX framework in neurorehabilitation prediction from clinical data and multimodal sensors. With the help of a hybrid sensor supervision objective, this proposed study identified an effective trade-off between the explainability-based attention regularization and prediction accuracy. The batch size was set to 128, with 10 consecutive epochs without validation. The Adam optimizer is utilized for training with a learning rate of 0.00005; weight decay of 0.0001; and β_1_ = 0.9 and β_2_ = 0.999. To effectively analyze the robustness of the Rehab-DRLX model under varied temporal segmentations with different dropout regularizations. The proposed study was benchmarked against various state-of-the-art works. In addition, the explainability model was evaluated using attention heatmaps and clinical alignment scores—both validated by clinicians.

### 4.1 Implementation details

The Rehab-DRLX framework was implemented within a multi-stage computational objective. The experiments were carried out in a system with an Intel Core i9-13900K processor, NVIDIA RTX 4090 GPU, and 64 GB DDR5 RAM with 24 GB VRAM. The operating system (OS) used was Ubuntu 22.04 LTS. Using Python 3.11 and its libraries, such as Open3D, Pandas, and NumPy, the data processing was performed. To perform formatting and data extraction, PyKinect2 and MATLAB R2023b were utilized. Using TensorFlow 2.15 and the Keras API, the reinforced representation learning (RRL) module was implemented. More specifically, using the TF agents, the reinforcement learning agents were trained alongside customized CNN encoders. The explainable prognosis transformer (XPT) module was designed using the Hugging Face Transformers library, which was mainly used for sensor data rather than text input. The mixed precision training and GPU acceleration were adopted from cuDNN 8.8.1 and NVIDIA Apex.

The average processing time for a single motion frame was approximately 18.7 ms, including CNN-based feature extraction, reinforcement learning-based embedding updates, and transformer-based prognosis inference. Considering that the Kinect v2 sensor operates at approximately 30 frames per second, the proposed framework satisfies real-time processing constraints. In addition, the end-to-end latency for generating a prognosis prediction for a rehabilitation segment was approximately 0.42 s, demonstrating that the proposed system can support near real-time clinical monitoring during rehabilitation sessions. The hyperparameters for the baseline models were initialized using the values reported in the respective studies, when available. For models where hyperparameter details were not explicitly provided, commonly used configurations were adopted to ensure stable training. The training process used the Adam optimizer with learning rates ranging between 1e-4 and 5e-5, a batch size of 128, and early stopping based on validation performance. All models were trained under identical computational conditions to ensure a fair comparison.

### 4.2 Evaluation metrics

To examine the Rehab-DRLX performance, this research employs classification, regression, and explainability metrics. Those metrics examine not only accuracy but also interpretability and clinical reliability based on the model output. The metrics include accuracy, F1-score, root mean square (RMSE), R-squared, attention alignment score (AAS), and Attention Entropy (AE). The mathematical Equations (21–25) for the performance metrics is provided as below:

- Accuracy: the accuracy examines the complete correctness of the model in effectively forecasting the outcomes. The formulation is provided as

$$\begin{array}{l} \text{Acc}=\frac{\text{TP}+\text{TN}}{\text{TP}+\text{TN}+\text{FP}+\text{FN}} \end{array}$$ (21)

From Equation 21, the true positive, true negative, false positive, and false negatives are denoted by TP, TN, FP, and FN, respectively.

- F1-score: the F1-score identified an effective trade-off between the recall and precision in terms of false positives and negatives, respectively. The formulation is provided as follows:

$$\begin{array}{l} \text{F}1\;=\;2\times \frac{\text{Precision }\times \text{ Recall}}{\text{Precision}+\text{Recall}} \end{array}$$ (22)

- RMS: the RMS is utilized when the outcome is a continuous recovery score. It also penalizes the larger errors, which makes it suitable for clinical applications as

$$\begin{array}{l} \text{RMSE}={\sqrt{\frac{1}{\text{n}}\sum_{\text{j}=1}^{\text{n}}\left({\text{y}}_{\text{j}}-{\hat{\text{y}}}_{\text{j}}\right)}}^{2} \end{array}$$ (23)

- R-squared: it measures how effectively the Rehab-DRLX provides the explainability in recovery outcomes. The formulation is provided as,

$${\text{R}}^{\text{2}}=1-\frac{\sum_{\text{j=1}}^{\text{n}}{\text{y}}_{\text{j}}-{\hat{\text{y}}}_{\text{j}}{)}^{2}}{\sum_{\text{j=1}}^{\text{n}}{\left({\text{y}}_{\text{j}}-\bar{\text{y}}\right)}^{2}}$$ (24)

- AAS: the AAS examines how the attention model effectively weights the clinically relevant features (i.e., annotated key points by the therapist). The formulation is provided by

$$\begin{array}{l} \text{AAS}=\frac{1}{\text{n}}\sum_{\text{j}=1}^{\text{n}}\sum_{\text{t}=1}^{\text{T}}1\left({\text{a}}_{\text{j},\text{t}}\in {\text{ℂ}}_{ℷ,\text{t}}\right) \end{array}$$ (25)

To obtain clinically relevant reference annotations for computing the attention alignment score (AAS), the rehabilitation motion sequences were independently reviewed by three certified physiotherapists with expertise in neurorehabilitation therapy. Each therapist examined the skeletal joint trajectories and temporal motion patterns to identify key rehabilitation phases and clinically significant movement segments. The annotation process focused on identifying critical time steps associated with meaningful therapeutic movements and recovery indicators. To ensure annotation reliability, inter-rater agreement was evaluated using Cohen's Kappa coefficient, which yielded a score of 0.81, indicating strong agreement among the therapists. The final ground-truth annotations were derived using a majority voting strategy, where the consensus labels from the therapists were used as the clinical reference for evaluating model attention alignment.

**Figure 3:**
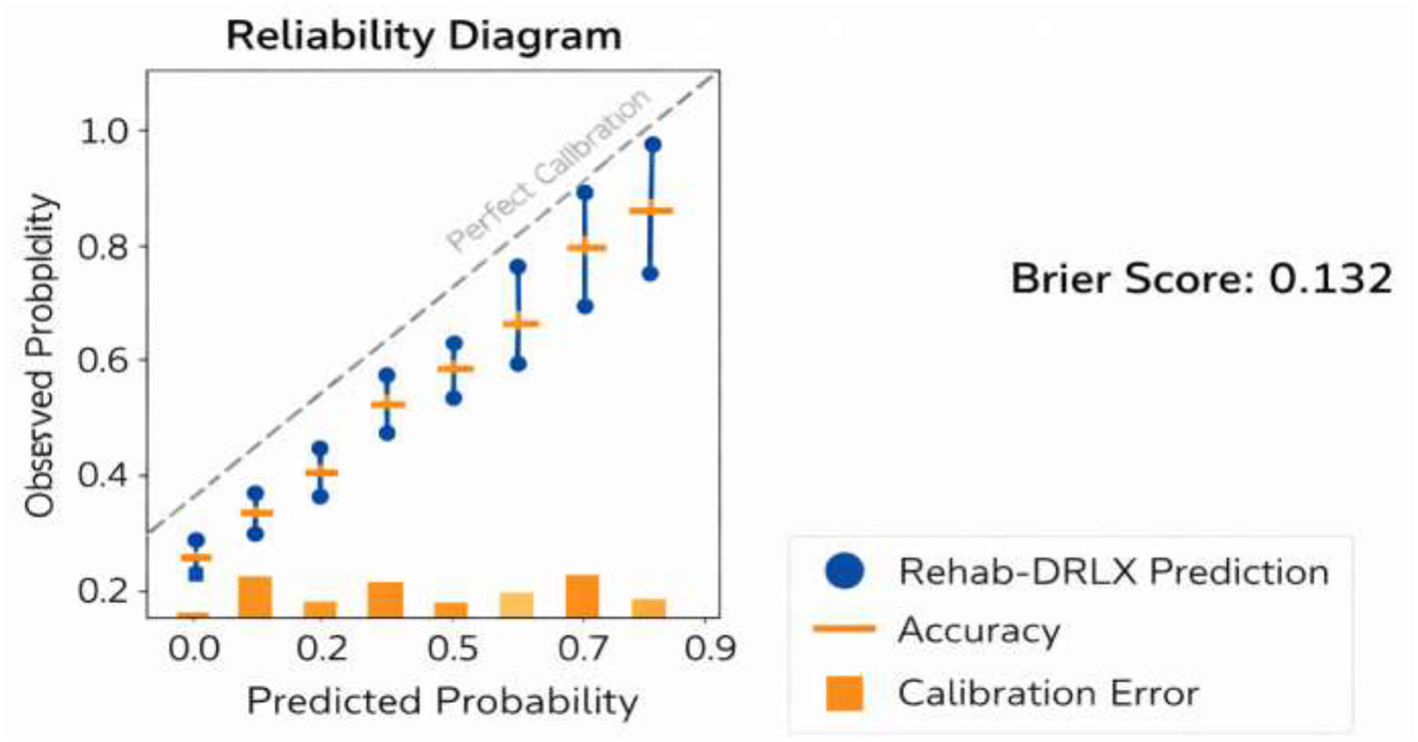
Reliability diagram of the Rehab-DRLX model showing predicted vs. observed probabilities.


- Attention entropy (AE): the AE computes the attention weights by suggesting the highlights of the model with an interpretable region of input (Figure 3). The formulation defined in Equation 26

$$\begin{array}{l} \text{A}{\text{E}}_{\text{j}}=-\sum_{\text{i}=1}^{\text{d}}{\text{α}}_{\text{ji}}\text{log}\left({\text{α}}_{\text{ji}}\right) \end{array}$$ (26)

In addition to traditional classification and regression metrics, this study also evaluates the calibration quality of probabilistic predictions produced by the Rehab-DRLX framework. Calibration analysis examines whether the predicted recovery probabilities match the observed outcomes. The reliability diagram visualizes the relationship between predicted probabilities and observed outcomes by grouping predictions into probability bins and comparing their predicted confidence with empirical accuracy. A well-calibrated model produces predictions that closely follow the diagonal reference line. The Brier score measures the mean squared difference between predicted probabilities and the actual outcome labels and is defined as:


$$\begin{array}{l} \text{BS}=\frac{1}{\text{N}}\sum_{\text{i}=1}^{\text{N}}{\left({\text{p}}_{\text{i}}-{\text{y}}_{\text{i}}\right)}^{2} \end{array}$$ (27)

**Figure 4:**
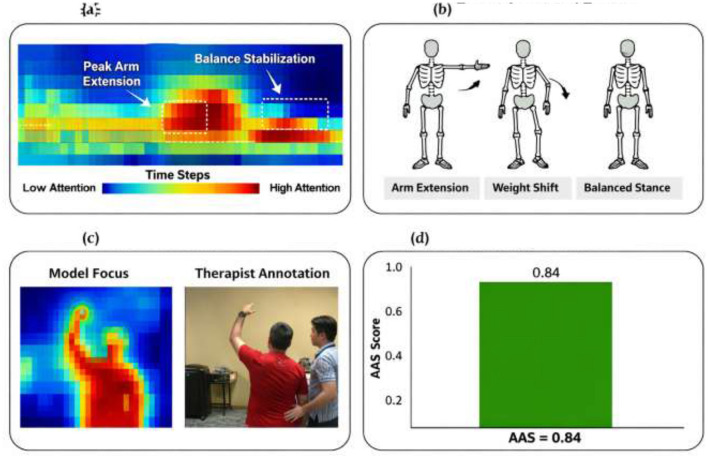
**(a)** Model attention heatmap. **(b)** Expert annotated events. **(c)** Key frame comparison. **(d)** Attention alignment score.

From Equation 27, the *p*_*i*_ represents the predicted probability of functional recovery and *y*_*i*_ denotes the corresponding ground-truth outcome. To demonstrate the interpretability of the proposed Rehab-DRLX framework, attention-based explanations were analyzed using the explainable prognosis transformer (XPT). The hierarchical attention mechanism produces temporal attention maps highlighting the rehabilitation frames and skeletal joint movements that contribute most significantly to the prognosis prediction. These attention distributions were visualized as heatmaps and compared with therapist-annotated key rehabilitation events (Figure 4).

The alignment between model attention and clinically relevant motion segments was quantitatively evaluated using the attention alignment score (AAS). Higher AAS values indicate stronger correspondence between the model's attention weights and therapist-identified rehabilitation events. The obtained AAS score indicates that the model consistently focuses on clinically meaningful phases of rehabilitation exercises, such as joint extension peaks and motion stabilization periods. Furthermore, the hierarchical attention layers allow examination of temporal feature attribution, enabling clinicians to identify which stages of the rehabilitation trajectory most strongly influence the predicted recovery outcome. These attention-based artifacts provide interpretable insights into the model's decision-making process of the model and improve transparency for clinical interpretation.

### 4.3 Comparative analysis

This section emphasizes the comparative analysis of the proposed and existing studies using five state-of-the-art methods, such as hybrid deep learning for post-stroke rehabilitation (HDL-PSR) (Bijalwan et al., 2023), machine learning-based brain-computer interface (ML-BCI) (Shao et al., 2023), graph-attentive convolutional LSTM network (GACL-Net) (Bunterngchit et al., 2025), TraxVBF (Zendehbad et al., 2025), and MSFormer (Zhao et al., 2025).

#### 4.3.1 Qualitative analysis

The conventional works on neurorehabilitation prognosis have seen a surge in hybrid deep learning models that focus on spatiotemporal representation and BCI. The HDL-PSR integrates CNNs and LSTMs to capture the dynamics of spatial and temporal information in post-stroke rehabilitation, which hinders interpretability, which is essential for adaptive clinical decision support. In a similar manner, the ML-BCI (Shao et al., 2023) combines the BCI in separate models but lacks in higher complexity due to over-reliance on a traditional machine learning pipeline and pre-segmented EEG dataset, which limits its adaptability in a real-world environment. The GACL-Net (Bunterngchit et al., 2025) used motor imagery categorization, achieving higher accuracy by combining a CNN and an LSTM; however, it was an offline setup and did not perform prognostic modeling. Different from that, the Rehab-DRLX designs a robust and combined model that is composed of CNN-DRL and can adaptively adjust the spatial features entrenched on feedback from patient progression states. In contrast to the TraxVBF (Zendehbad et al., 2025) model that integrates LSTM and transformer from EMG signal processing, but does not model explainability. The Rehab-DRLX combines XPT, which is armed with hierarchical attention and positional encoders. By introducing explainability, the clinical experience is enhanced by effectively examining key neurological events. The MSFormer (Zhao et al., 2025) employs motor imagery but did not attain generalizability to handle multimodal data; the Rehab-DRLX has a novel design to handle multimodal rehabilitation trajectories and complex data, which makes it clinically deployable.

It is important to note that several existing rehabilitation models reported in the literature primarily focus on classification-oriented tasks, such as motor imagery recognition or rehabilitation activity categorization, whereas the proposed Rehab-DRLX framework performs prognosis-oriented prediction, including both categorical outcome prediction and continuous recovery score estimation. To ensure consistent comparison, the baseline architectures were reimplemented and evaluated using the same dataset, preprocessing pipeline, training configuration, and validation protocol used for the proposed model. Performance was assessed using a combination of classification metrics (accuracy, precision, recall, and F1-score) and regression metrics [RMSE and mean absolute error (MAE)] to capture both categorical decision quality and continuous prognosis estimation accuracy. This unified evaluation protocol enables a fair comparison while reflecting the dual predictive capability of the proposed neurorehabilitation prognosis framework.

#### 4.3.2 Quantitative analysis

**Figure 5:**
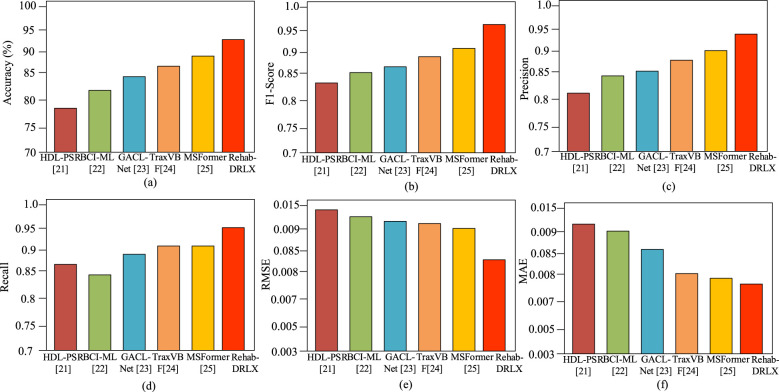
**(a)** Accuracy comparison of proposed studies vs. existing studies, **(b)** F1-score comparison of proposed studies vs. existing studies, **(c)** precision comparison of proposed studies vs. existing studies, **(d)** recall comparison of proposed studies vs. existing studies, **(e)** RMSE comparison of proposed studies vs. existing studies, and **(f)** MAE comparison of proposed studies vs. existing studies.

In terms of quantitative analysis, the HDL-PSR (Bijalwan et al., 2023) ensures better results in classification, with an accuracy of 89.5% from hybrid deep learning (HDL) models named CNN-LSTM. The ML-BCI (Shao et al., 2023) and GACL-Net (Bunterngchit et al., 2025) gain better classification accuracy of 90.2 and 91.2% in stroke rehabilitation tasks. Further, the TraxVBF (Zendehbad et al., 2025) achieves better classification performance of 94.1% by employing LSTM and transformer layers. The MS-Former achieves 93.3% classification accuracy, as it only employs a transformer and does not consider multimodal data. The proposed Rehab-DRLX was examined among the multimodal datasets, which include clinical progression logs and Kinect-entrenched motion data, resulting in RMSE, F1-score, and accuracy of 0.082, 94.7%, 95.3%, respectively, in regression-entrenched prognosis tasks. In addition, the model achieves an *R*^2^ of 0.91 with higher proportional clinical recovery scores. The higher AAS of 0.78 reflects the better correspondence in feature learning than the existing studies. Table 1 and Figures 5a–f present the quantitative comparison among the proposed and existing studies.

**Table 1:** Comparative analysis of proposed studies vs. existing studies.

Model	Accuracy (%)	F1-score	Precision	Recall	RMSE	MAE
HDL-PSR (Bijalwan et al., 2023)	86.7	0.84	0.81	0.87	0.126	0.093
ML-BCI (Shao et al., 2023)	88.2	0.85	0.83	0.86	0.112	0.089
GACL-Net (Bunterngchit et al., 2025)	89.5	0.86	0.84	0.87	0.109	0.085
TraxVBF (Zendehbad et al., 2025)	90.1	0.88	0.86	0.90	0.104	0.079
MSFormer (Zhao et al., 2025)	91.2	0.89	0.88	0.90	0.097	0.074
**Rehab-DRLX (ours)**	**94.6**	**0.93**	**0.91**	**0.94**	**0.082**	**0.061**

The bold values represent the results of the proposed method.

To assess whether the observed improvements of the proposed model are statistically meaningful, statistical significance analysis was conducted across the cross-validation folds. Specifically, a paired *t*-test was performed between the proposed Rehab-DRLX framework and each baseline model using the evaluation metrics obtained from the cross-validation experiments. The statistical analysis confirmed that the improvements achieved by the proposed framework are statistically significant (*p* < 0.05) across the evaluated performance metrics, demonstrating the robustness and reliability of the proposed approach compared to existing methods.

### 4.4 Ablation study

To show the contribution of each component of the proposed Rehab-DRLX model, we conduct an ablation study focusing on three proposed modules: RRL, XPT, and the dual supervision objective. Below, we explain the details of the modules' impact.

#### 4.4.1 Impact of RRL module

**Figure 6:**
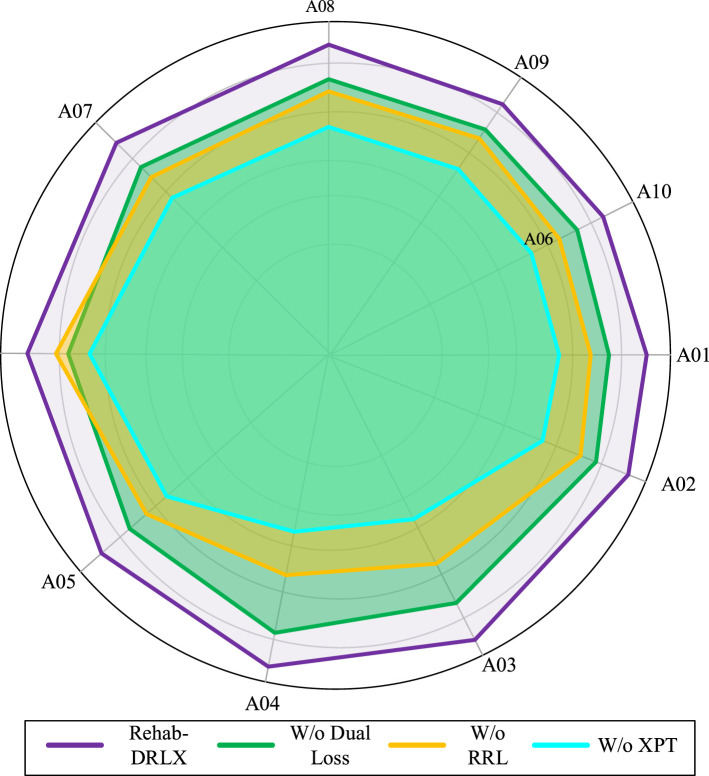
Radar plot visualization of Rehab-DRLX model.

By removing the DRL and utilizing only a CNN encoder for spatial feature extraction, it is observed that there is 6.2% of accuracy drop, with a predominant increase in RMSE from 0.0082 to 0.119. Those performance drop changes show the importance of DRL for temporal change extraction in patient recovery. Without the DRL agent for policy guidance, entrenched on functional outcomes. More clearly, the model had the capability to capture the time-dependent and nuanced rehabilitation variations. In addition to that, the sensitivity of the model to early stage recovery indicators is reduced, which leads to diminished long-term prognosis estimation. Table 2 and Figure 6 show the ablation analysis quantitatively and diagrammatically.

**Table 2:** Quantitative comparison of ablation analysis.

Model variant	Accuracy	F1-score	Precision	Recall	RMSE	MAE
W/o DRL agent (no RL loop)	89.3	0.87	0.85	0.88	0.115	0.091
W/o CNN embeddings	86.7	0.84	0.82	085	0.129	0.102
W/o transformer	88.1	0.85	0.84	0.86	0.122	0.097
W/o dual supervision objective	90.5	0.88	0.87	0.88	0.108	0.083
W/o attention gates	91.2	0.89	0.87	0.90	0.104	0.078
W/o clinical contextual positional encoding	92.0	0.90	0.88	0.91	0.097	0.073
**Rehab-DRLX (ours)**	**94.6**	**0.93**	**0.91**	**0.94**	**0.082**	**0.061**

The bold values represent the results of the proposed method.

#### 4.4.2 Impact of XPT module

Afterward, we examine the model without the transformer-entrenched XPT. In the replacement, we use a conventional BiLSTM for processing sequential sensor and clinical embeddings, which results in a 4.8% lower F1-score and decreased explainability, measured by AAS dropping from 0.78 to 0.53. The XPTs' hierarchical attention gates and clinical contextual positional encoders enable better resolution interpretability, which allows the model to effectively localize the importance of the clinical time points. Without XPT, the meaningful recovery factors were not traced by the clinicians, which shows the lower specificity in delayed neurological response. Those findings validate that XPT provides transparency and predictive power, which are highly essential for clinical adoption.

#### 4.4.3 Impact of dual supervision objective

To examine the performance of the dual supervision objective, this research replaced it with a single loss function that minimized only the prediction error, thereby decreasing both accuracy and interpretability. In a specific manner, the explainability-based attention regularization loss generated attention maps that reduced the AE from 0.34 to 0.21. Without that, the attention layers weaken the ability of the model to highlight the relevant input signals. In addition to that, the RMSE also increased significantly with lesser confidence calibration, which indicates that the interpretability constraints indirectly enhance the model robustness and trustworthiness of the prognosis prediction.

### 4.5 Explainability case studies and visualization

To further demonstrate the interpretability capability of the proposed Rehab-DRLX framework, this section presents several explainability artifacts that illustrate how the model identifies clinically meaningful rehabilitation patterns. The visual explanations include temporal attention heatmaps and SHAP-based feature attribution analysis.

#### 4.5.1 Temporal–joint attention heatmap analysis

The explainable prognosis transformer (XPT) generates attention distributions across rehabilitation time steps and skeletal joints. These attention weights indicate which temporal segments and joint movements contribute most strongly to the predicted recovery outcome. For interpretability analysis, we selected three representative rehabilitation cases from the test dataset. The attention weights were visualized as heatmaps, with rows representing skeletal joints and columns representing temporal frames of the rehabilitation sequence.

**Figure 7:**
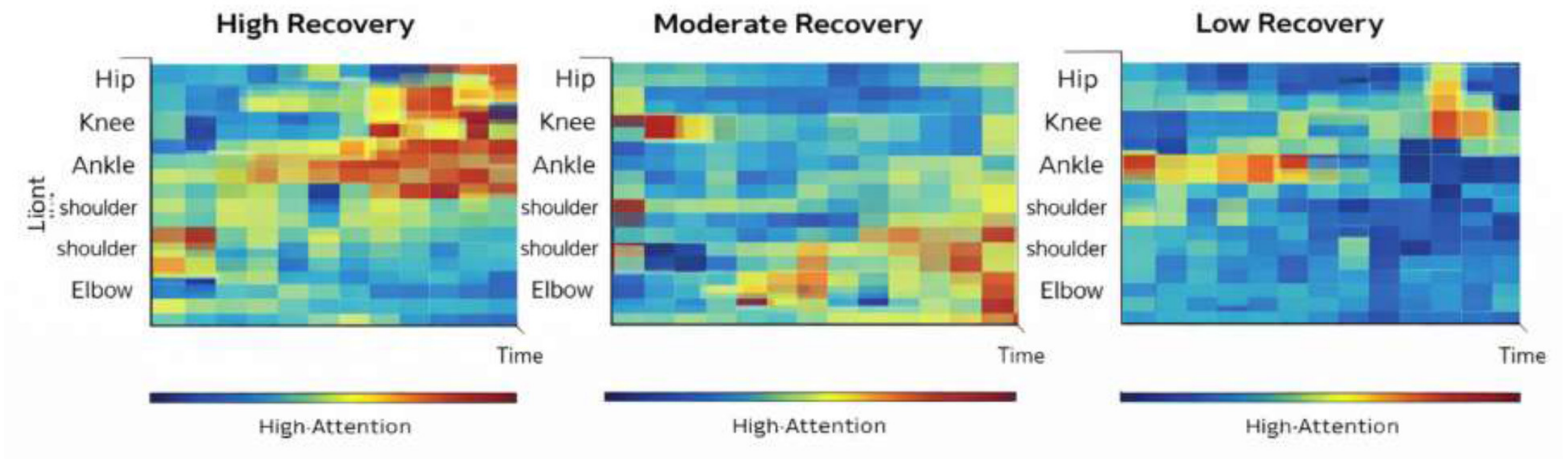
Visualization Heatmap of High recovery, Moderate recovery, Low recovery.

- Case 1—high recovery outcome: the model strongly focuses on knee extension, hip stabilization, and ankle coordination during the peak rehabilitation phase.
- Case 2—moderate recovery outcome: attention is distributed across multiple joints but shows reduced focus on stability phases.
- Case 3—low recovery outcome: the model emphasizes irregular motion patterns and unstable joint trajectories (Figure 7).

#### 4.5.2 SHAP-based feature attribution analysis

**Figure 8:**
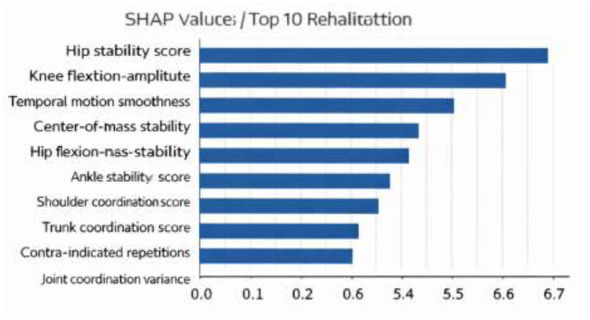
SHAP Analysis of Feature Attribution.

To further analyze feature-level contributions, SHapley Additive exPlanations (SHAP) were applied to estimate the influence of individual features on the predicted recovery probability. The SHAP analysis quantifies how each input feature increases or decreases the predicted outcome. The most influential features include the hip joint stability score, knee flexion–extension amplitude, temporal motion smoothness, center-of-mass stability, and joint coordination variance (Figure 8).

#### 4.5.3 Correct vs. incorrect prediction case analysis

To examine the reliability of the model explanations, we analyzed representative correct and incorrect prediction cases from the test dataset. Each case was reviewed alongside clinician annotations of key rehabilitation phases.

**Figure 9:**
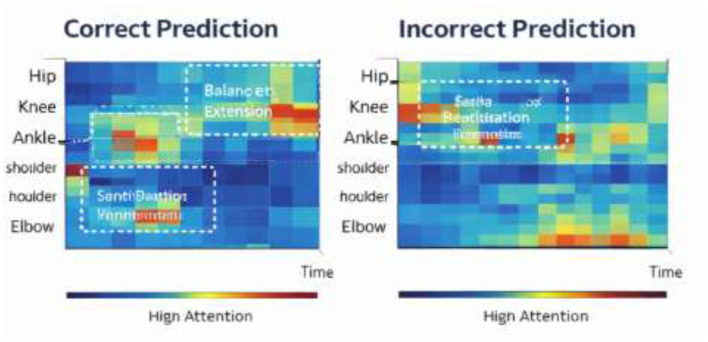
Illustration of Prediction Performance Heatmap.

- Correct prediction case: the attention map aligns closely with clinically relevant phases such as peak joint extension and balance stabilization, indicating strong agreement between the model and therapist interpretation.
- Incorrect prediction case: in rare cases, the model focuses on secondary motion segments, which may lead to inaccurate prognosis estimation. These cases highlight potential areas for future model improvement (Figure 9).

## 5 Conclusion

In conclusion, this research proposes a Rehab-DRLX, a novel interpretable deep learning model for effective prognosis in neurorehabilitation tasks. By integrating DRL and Explainable transformers, the proposed model robustly extracts spatiotemporal recovery patterns by aligning forecasting with clinical insights. Across extensive experiments, the proposed model outperforms existing studies in terms of interpretability and accuracy. We then also validate the proposed model's efficacy in a complex patient recovery trajectory. By participating in dynamic reinforcement learning with an attention mechanism that not only predicts the functional outcomes but also provides clinicians with interpretable and trusted decision-making. This dual supervision makes the model effective to deploy in real-world neurorehabilitation settings where the interpretability and precision are crucial.

As a future initiative, this research aims to extend the framework to include interpretability across diverse patient populations and rehabilitation protocols, paving the way for cognitive and patient-based recovery support systems.

## Data Availability

The raw data supporting the conclusions of this article will be made available by the authors, without undue reservation.
